# Synthesis of Scutellarein Derivatives with a Long Aliphatic Chain and Their Biological Evaluation against Human Cancer Cells

**DOI:** 10.3390/molecules23020310

**Published:** 2018-02-01

**Authors:** Guanghui Ni, Yanling Tang, Minxin Li, Yuefeng He, Gaoxiong Rao

**Affiliations:** 1 College of Pharmaceutic Science, Yunnan University of Traditional Chinese Medicine, Kunming 650500, China; niguanghui@ynutcm.edu.cn (G.N.); m18487302692@163.com (Y.T.); lmx04087@163.com (M.L.); 2 Engineering Laboratory for National Healthcare Theories and Products of Yunnan Province, Yunnan University of Traditional Chinese Medicine, Kunming 650500, China; 3 School of Public Health, Kunming Medical University, Kunming 650500, China

**Keywords:** scutellarein, scutellarin, flavonoid, cancer, anti-proliferation, long aliphatic chain

## Abstract

Scutellarin is the major active flavonoid extracted from the traditional Chinese herbal medicine *Erigeron breviscapus* (Vant.) Hand-Mazz., which is widely used in China. Recently, accumulating evidence has highlighted the potential role of scutellarin and its main metabolite scutellarein in the treatment of cancer. To explore novel anticancer agents with high efficiency, a series of new scutellarein derivatives with a long aliphatic chain were synthesized, and the antiproliferative activities against Jurkat, HCT-116 and MDA-MB-231 cancer cell lines were assessed. Among them, compound **6a** exhibited the strongest antiproliferative effects on Jurkat (IC_50_ = 1.80 μM), HCT-116 (IC_50_ = 11.50 μM) and MDA-MB-231 (IC_50_ = 53.91 μM). In particular, **6a** even showed stronger antiproliferative effects than the positive control NaAsO_2_ on Jurkat and HCT-116 cell lines. The results showed that a proper long aliphatic chain enhanced the antiproliferative activity of scutellarein.

## 1. Introduction

**Figure 1:**
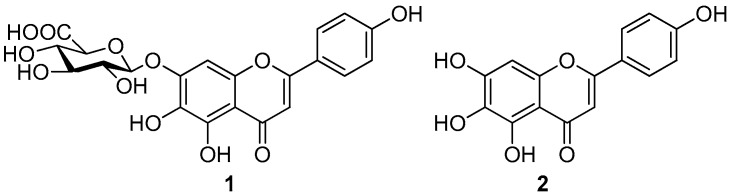
Structures of scutellarin (**1**) and scutellarein (**2**).

Cancer is the most threatening and difficult-to-treat disease. Traditional Chinese herbal medicines have been applied for the treatment of cancers for many years [1]. Compounds isolated from traditional Chinese herbs exhibit pharmacological activities [2] and have been recognized as valuable resources for the development of novel anti-cancer drugs [3]. Scutellarin (**1**, Figure 1) is the major active flavonoid extracted from the traditional Chinese herbal medicine Erigeron breviscapus (Vant.) Hand-Mazz., which is used clinically in cardiovascular diseases [4]. Recently, accumulating evidence has highlighted the potential role of scutellarin and its main metabolite scutellarein (**2**, Figure 1) [5] in the treatment of cancer [6,7,8]. Nevertheless, low stability and poor lipophilicity of scutellarin and scutellarein limited their further application [9]. Derivatives have been synthesized to overcome these disadvantages and increase biological activity [10,11,12]. We report herein the synthesis of a series of scutellarein derivatives with a long aliphatic chain and their evaluation as anti-cancer agents.

## 2. Results and Discussion

**Scheme 1:**
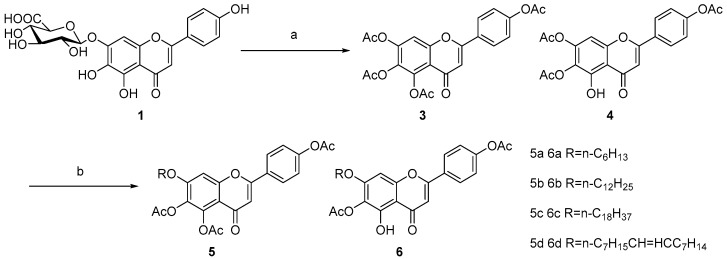
Reagents and conditions: a. Ac_2_O, pyridine, DMAP, reflux, 86%; b. RBr, K_2_CO_3_, DMF, 53–72%.

The methodologies for preparing the target scutellarein derivatives are outlined in Scheme 1. Scutellarin (**1**) was reacted with acetic anhydride and catalytic 4-*N*,*N*-dimethylaminopyridine (DMAP) in pyridine under reflux to afford 5,6,7,4′-*O*-tetraacetyl scutellarein (**3**) in 54% yield and 6,7,4′-*O*-triacetyl scutellarein (**4**) in 23% yield. 6,7,4′-*O*-triacetyl scutellarein (**4**) is assigned its structure based on ^1^H-NMR analysis. Following literature protocols [13,14], the assignment of the single free phenol hydroxyl group utilized the narrow peak shape and high chemical shift (12.90 ppm, CDCl_3_) of the hydroxyl proton. The single free phenol hydroxyl group is at the C-5 position, and can form a hydrogen bond with the carbonyl group at C-4 position [15].

**Figure 2:**
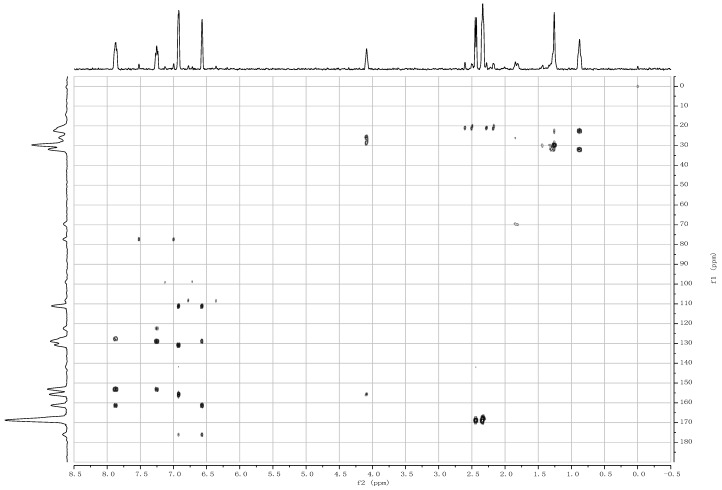
HMBC spectrum of **5d**.

**Figure 3:**
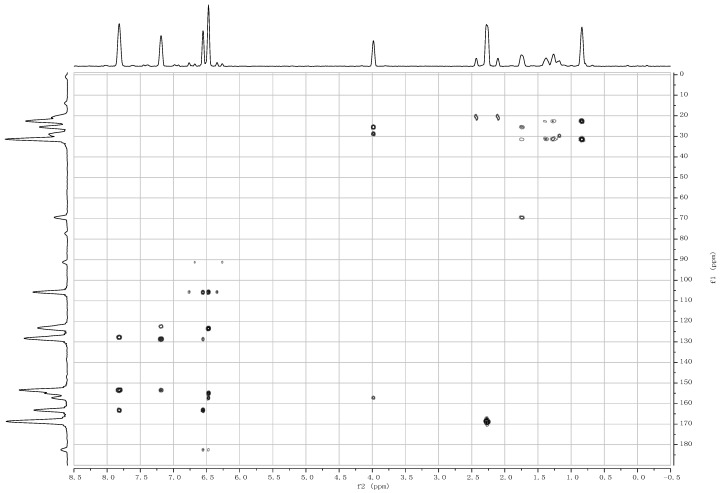
HMBC spectrum of **6a**.

**Figure 4:**
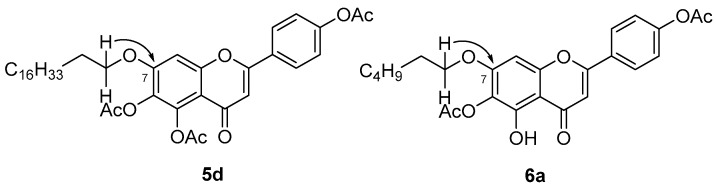
Key HMBC correlations of compounds of **5d** and **6a**.

**3** and **4** are converted to 7-*O*-aliphatic-5,6,4′-*O*-triacetylscutellarein (**5**) and 7-*O*-aliphatic-6,4′-*O*-diacetylscutellarein (**6**) by reacting with RBr using K_2_CO_3_ in *N*,*N*-dimethylformamide (DMF). The position of aliphatic chain connected with scutellarein was clarified by using Hetero-nuclear Multiple-Bond Connectivity (HMBC). The triplet at 4.08 ppm in the spectrum of **5d** (Figure 2) belongs to the OCH_2_ protons of the aliphatic chain. A cross peak is observed at 155.68 ppm, and belongs to C-7. Similarly, the triplet at 4.06 ppm in the spectrum of **6a** (Figure 3) belongs to the OCH_2_ protons of the aliphatic chain. A cross peak is observed at 157.30 ppm, and belongs to C-7. The OCH_2_ protons of the aliphatic chain were correlated with C-7 as shown in Figure 4 indicated that the aliphatic chain is bonded to the C-7 position [16].

The biological activities of 7-*O*-aliphatic scutellarein derivatives **5a**–**d** and **6a**–**d** in inhibiting cancer cell line proliferation were assayed in vitro using CellTiter 96^®^ AQueous One Solution Cell Proliferation Assay (MTS) according to the previously reported method [17]. Anticancer drug sodium arsenite (NaAsO_2_) [18,19] was used as a positive control. Unexpectedly, most of the compounds were not able to inhibit the cancer cell proliferation at concentrations ≤100 μM, as shown in Table 1. However, the compounds **5a**, **5b**, **6a** and **6b** displayed anti-proliferation activity at concentrations ≤100 μM, and their activities were higher than both scutellarin and scutellarein. In particular, **6a** even showed stronger antiproliferative effects than the positive control NaAsO_2_ on Jurkat and HCT-116 cell lines. The results showed that the aliphatic chain enhanced the anti-proliferation activity. However, the anti-proliferation activity is not stronger when the aliphatic chain is longer. A proper long aliphatic chain is beneficial to enhancing the anti-proliferative activity of scutellarein. **6a** exhibited the strongest antiproliferative effects on Jurkat (IC_50_ = 1.80 μM), HCT-116 (IC_50_ = 11.50 μM) and MDA-MB-231 (IC_50_ = 53.91 μM). In addition, **6b** with a free phenol hydroxyl group at C-5 position exhibited stronger anti-proliferative effects than **5b**. These results indicate that the hydroxyl group at C-5 of scutellarein derivatives may be important anti-proliferative activity.

## 3. Materials and Methods

Reagents and solvents were obtained from commercial suppliers and used without further purification unless stated otherwise. Pyridine was dried by boiling with CaH_2_ prior to distillation and stored over KOH, DMF (*N*,*N*-dimethylformamide) was stored over 4 Å molecular sieves. Reactions were monitored by thin-layer chromatography (TLC), and performed on 0.2 mm silica gel GF254 plates using UV light as the visualizing agent. Chromatographic purification of products was accomplished using forced-flow chromatography on 300–400 mesh silica gel. The ^1^H-NMR (400 MHz, CDCl_3_) and ^13^C-NMR (100 MHz, CDCl_3_) spectra were measured on a Bruker AV 400 MHz (Bruker, Karlsruhe, Germany) in CDCl_3_ as solvent, using (TMS) as an internal standard, and chemical shifts are expressed as δ ppm.

*5,6,7,4′-O-Tetraacetyl scutellarein* (**3**) and *6,7,4′-O-triacetyl scutellarein* (**4**) Scutellarin (10 g, 21.6 mmol) was dissolved into the Ac_2_O/Pyridine(50 mL, 3:2, *v*/*v*) solution and reacted reflux for 4 h. After the reaction was completed (monitored by TLC), the solvent was removed, and the resulting residue was then purified by column chromatography on silica gel using 50% ethyl acetate in petroleum ether as eluent to afford **3** (5.3 g, 11.7 mmol, 54%) and **4** (2.1 g, 5.1 mmol, 23%).

*5,6,7,4′-O-Tetraacetyl scutellarein* (**3**) yellow solid. ^1^H-NMR (CDCl_3_, 400 MHz) δ: 7.88 (d, 2H, *J* = 8.4 Hz, Ar′-H-2,6), 7.49 (s, 1H, Ar-H-8), 7.27 (d, 2H, *J* = 6.0 Hz, Ar′-H-3,5); 6.62 (s, 1H, Ar-H-3); 2.44 (s, 3H, COCH_3_), 2.35 (s, 9H, COCH_3_ × 3).

*6,7,4′-O-Triacetyl scutellarein* (**4**) yellow solid, m.p. 261–264 °C. ^1^H-NMR (CDCl_3_, 400 MHz) δ: 12.90 (s, 1H, OH), 7.91 (d, 2H, *J* = 7.8 Hz, Ar′-H-2,6), 7.29 (d, 2H, *J* = 7.9 Hz, 2H, Ar′-H-3,5), 6.96 (s, 1H, Ar-H-3), 6.70 (s, 1H, Ar-H-8), 2.37 (s, 3H, COCH_3_), 2.35 (s, 6H, COCH_3_ × 2). 

General Procedure for Preparation of 7-*O*-aliphatic-5,6,4′-*O*-triacetyl scutellarein (5) and 7-*O*-aliphatic-6,4′-*O*-diacetyl scutellarein (6). 

To a solution of compound **3** or **4** (1 mmol) in anhydrous DMF (10 mL) was added K_2_CO_3_ (691 mg, 5 mmol) and RX (1.5 mmol), then the reaction mixture was stirred overnight at room temperature. The mixture was poured into water (50 mL), and then extracted with ethyl acetate (50 mL × 3), the organic layer was washed with brine (100 mL), dried over Na_2_SO_4_, filtered and concentrated under reduced pressure. The crude material that appeared was then purified by column chromatography on silica gel using 20% ethyl acetate in petroleum ether as eluent to afford **5** or **6**. 

*7-O-Hexyl-5,6,4′-O-triacetylscutellarein* (**5a**); yellow solid; yield 72%; ^1^H-NMR (400 MHz, CDCl_3_) δ 7.87 (d, *J* = 8.8 Hz, 2H, Ar′-H-2,6), 7.26 (d, *J* = 8.8 Hz, 2H, Ar′-H-3,5), 6.92 (s, 1H, Ar-H-3), 6.57 (s, 1H, Ar-H-8), 4.09 (t, *J* = 6.5 Hz, 2H, OCH_2_), 2.44 (s, 3H, COCH_3_), 2.34 (s, 3H, COCH_3_), 2.33 (s, 3H, COCH_3_), 1.88–1.76 (m, 2H, CH_2_), 1.49–1.42 (m, 2H, CH_2_), 1.39–1.31 (m, 4H, CH_2_ × 2), 0.92 (t, *J* = 6.7 Hz, 3H, CH_3_). ^13^C-NMR (100 MHz, CDCl_3_) δ 176.23, 168.90, 168.73, 167.83, 161.23, 155.79, 155.69, 153.17, 141.77, 130.86, 128.94, 127.47, 122.32, 111.09, 108.26, 98.70, 69.63, 31.36, 28.69, 25.44, 22.52, 21.12, 20.85, 20.10, 13.94.

*7-O-Dodecyl-5,6,4′-O-triacetylscutellarein* (**5b**) yellow solid; yield 72%; ^1^H-NMR (400 MHz, CDCl_3_) δ 7.86 (d, *J* = 8.4 Hz, 2H, Ar′-H-2,6), 7.25 (d, *J* = 8.4 Hz, 2H, Ar′-H-3,5), 6.92 (s, 1H, Ar-H-3), 6.56 (s, 1H, Ar-H-8), 4.08 (t, *J* = 6.4 Hz, 2H, OCH_2_), 2.44 (s, 3H, COCH_3_), 2.34 (s, 3H, COCH_3_), 2.33 (s, 3H, COCH_3_), 1.91–1.71 (m, 2H, CH_2_), 1.49–1.40 (m, 2H, CH_2_), 1.38–1.23 (m, 16H, CH_2_ × 8), 0.88 (t, *J* = 6.7 Hz, 3H, CH_3_). ^13^C-NMR (100 MHz, CDCl_3_) δ 176.20, 168.89, 168.72, 167.82, 161.21, 155.77, 155.68, 153.16, 141.75, 130.85, 128.91, 127.46, 122.31, 111.06, 108.22, 98.71, 69.62, 31.91, 29.69, 29.65, 29.63, 29.56, 29.53, 29.33, 29.23, 28.74, 25.78, 22.67, 21.12, 20.86, 20.11, 14.10.

*7-O-Octadecanyl-5,6,4′-O-triacetylscutellarein* (**5c**) yellow solid; yield 57%; ^1^H-NMR (400 MHz, CDCl_3_) δ 7.87 (d, *J* = 8.4 Hz, 2H, Ar′-H-2,6), 7.25 (d, *J* = 8.4 Hz, 2H, Ar′-H-3,5), 6.92 (s, 1H, Ar-H-3), 6.57 (s, 1H, Ar-H-8), 4.09 (t, *J* = 6.5 Hz, 2H, OCH_2_), 2.44 (s, 3H, COCH_3_), 2.34 (s, 3H, COCH_3_), 2.33 (s, 3H, COCH_3_), 1.91–1.74 (m, 2H, CH_2_), 1.51–1.39 (m, 2H, CH_2_), 1.26 (s, 28H, CH_2_ × 14), 0.88 (t, *J* = 6.7 Hz, 3H, CH_3_). ^13^C-NMR (100 MHz, CDCl_3_) δ 176.22, 168.90, 168.73, 167.84, 161.24, 155.79, 155.70, 153.17, 141.78, 130.87, 128.96, 127.47, 122.33, 111.09, 108.28, 98.69, 69.63, 31.91, 29.69, 29.65, 29.57, 29.53, 29.34, 29.32, 29.23, 28.75, 25.79, 22.67, 21.13, 20.85, 20.11, 14.08.

*7-O-Oleyl-5,6,4′-O-triacetylscutellarein* (**5d**) yellow solid; yield 70%; ^1^H-NMR (400 MHz, CDCl_3_) δ 7.86 (d, *J* = 8.8 Hz, 2H, Ar′-H-2,6), 7.24 (d, *J* = 8.8 Hz, 2H, Ar′-H-3,5), 6.91 (s, 1H, Ar-H-3), 6.56 (s, 1H, Ar-H-8), 5.43–5.30 (m, 2H, CH × 2), 4.08 (t, *J* = 6.4 Hz, 2H, OCH_2_), 2.44 (s, 3H, COCH_3_), 2.33 (s, 3H, COCH_3_), 2.33 (s, 3H, COCH_3_), 2.08–1.95 (m, 4H, CH_2_ × 2), 1.88–1.77 (m, 2H, CH_2_), 1.50–1.40 (m, 2H, CH_2_), 1.39–1.18 (m, 20H, CH_2_ × 10), 0.87 (t, *J* = 6.7 Hz, 3H, CH_3_). ^13^C-NMR (100 MHz, CDCl_3_) δ 176.18, 168.88, 168.71, 167.80, 161.21, 155.76, 155.68, 153.17, 141.76, 130.86, 130.02, 129.74, 128.92, 127.45, 122.31, 111.08, 108.24, 98.70, 69.60, 31.89, 29.75, 29.68, 29.52, 29.44, 29.31, 29.21, 28.75, 27.23, 27.20, 25.79, 22.67, 21.11, 20.85, 20.10, 14.09.

*7-O-Hexyl-6,4′-O-diacetylscutellarein* (**6a**) yellow solid; yield 72%; ^1^H-NMR (400 MHz, CDCl_3_) δ 12.72 (s, 1H, OH), 7.89 (d, *J* = 8.8 Hz, 1H), 7.26 (d, *J* = 8.8 Hz, 2H), 6.63 (s, 1H), 6.54 (s, 1H), 4.06 (t, *J* = 6.5 Hz, 2H), 2.35 (s, 3H), 2.34 (s, 3H), 1.96–1.73 (m, 2H), 1.51–1.40 (m, 2H), 1.39–1.30 (m, 4H), 1.29–1.19 (m, 3H), 0.97–0.87 (m, 3H). ^13^C-NMR (100 MHz, CDCl_3_) δ 182.42, 168.84, 168.43, 163.30, 157.30, 154.91, 153.48, 152.60, 128.74, 127.66, 123.55, 122.41, 105.85, 105.72, 91.29, 69.44, 31.39, 29.68, 28.76, 25.44, 22.53, 21.11, 20.21, 13.95.

*7-O-Dodecyl-6,4′-O-diacetylscutellarein* (**6b**) yellow solid; yield 68%; ^1^H-NMR (400 MHz, CDCl_3_) δ 12.72 (s, 1H, OH), 7.90 (d, *J* = 8.8 Hz, 2H, Ar′-H-2,6), 7.27 (d, *J* = 8.1 Hz, 2H, Ar′-H-3,5), 6.64 (s, 1H, Ar-H-3), 6.56 (s, 1H, Ar-H-8), 4.07 (t, *J* = 6.5 Hz, 2H, OCH_2_), 2.36 (s, 3H, COCH_3_), 2.35 (s, 3H, COCH_3_), 1.96–1.70 (m, 2H, CH_2_), 1.51–1.39 (m, 2H, CH_2_), 1.39–1.14 (m, 16H, CH_2_ × 8), 0.88 (t, *J* = 6.7 Hz, 3H, CH_3_). ^13^C-NMR (100 MHz, CDCl_3_) δ 182.44, 168.84, 168.44, 163.31, 157.31, 154.93, 153.47, 152.63, 128.78, 127.67, 123.58, 122.42, 105.88, 105.77, 91.29, 69.45, 31.91, 29.69, 29.65, 29.63, 29.57, 29.54, 29.34, 29.25, 28.81, 25.78, 22.67, 21.12, 20.23, 14.08.

*7-O-Octadecanyl-6,4′-O-diacetylscutellarein* (**6c**) yellow solid; yield 53%; ^1^H-NMR (400 MHz, CDCl_3_) δ 7.83 (d, *J* = 8.8 Hz, 2H, Ar′-H-2,6), 7.20 (d, *J* = 8.5 Hz, 2H, Ar′-H-3,5), 6.57 (s, 1H, Ar-H-3), 6.49 (s, 1H, Ar-H-8), 3.99 (t, *J* = 6.5 Hz, 2H, OCH_2_), 2.29 (s, 3H, COCH_3_), 2.27 (s, 3H, COCH_3_), 1.80–1.71 (m, 2H, CH_2_), 1.42–1.33 (m, 2H, CH_2_), 1.32–1.12 (m, 28H, CH_2_ × 14), 0.81 (t, *J* = 6.7 Hz, 6H, CH_3_). ^13^C-NMR (100 MHz, CDCl_3_) δ 182.43, 168.83, 168.42, 163.31, 157.30, 154.92, 153.48, 152.63, 130.02, 129.74, 128.77, 127.67, 122.41, 105.88, 105.76, 91.28, 69.43, 31.89, 29.77, 29.74, 29.69, 29.65, 29.52, 29.44, 29.32, 29.30, 29.22, 28.82, 27.23, 27.20, 25.79, 22.67, 21.12, 20.23, 14.08.

*7-O-Oleyl-6,4′-O-diacetylscutellarein* (**6d**) yellow solid; yield 59%; ^1^H-NMR (400 MHz, CDCl_3_) δ 12.72 (s, 1H, OH), 7.90 (d, *J* = 8.8 Hz, 2H, Ar′-H-2,6), 7.27 (d, *J* = 8.8 Hz, 2H, Ar′-H-3,5), 6.64 (s, 1H, Ar-H-3), 6.55 (s, 1H, Ar-H-8), 5.47–5.16 (m, 2H, CH × 2), 4.06 (t, *J* = 6.5 Hz, 2H, OCH_2_), 2.36 (s, 3H, COCH_3_), 2.34 (s, 3H, COCH_3_), 2.09–1.92 (m, 4H, CH_2_ × 2), 1.87–1.76 (m, 2H, CH_2_), 1.51–1.40 (m, 2H, CH_2_), 1.40–1.21 (m, 20H, CH_2_ × 10), 0.88 (t, *J* = 6.7 Hz, 3H, CH_3_). ^13^C-NMR (100 MHz, CDCl_3_) δ 182.43, 168.83, 168.42, 163.31, 157.30, 154.92, 153.48, 152.63, 130.02, 129.74, 128.77, 127.67, 122.41, 105.88, 105.76, 91.28, 69.43, 31.89, 29.77, 29.74, 29.69, 29.65, 29.52, 29.44, 29.32, 29.30, 29.22, 28.82, 27.23, 27.20, 25.79, 22.67, 21.12, 20.23, 14.08.

### Cell Culture and Treatment

Cells were plated in 96-well plates (Jet Bio-Filtration, Guangzhou, China, 5000 cells/well in 100 μL) and grown for 24 h at 37 °C before being treated with various concentrations of the tested compounds (from 1 to 200 μM). After 48 h of incubation, CellTiter 96^®^ AQueous One Solution Cell Proliferation Assay (MTS) solution (Promega, Madison, WI, USA) was added to each well, and the plates were further incubated for 1 h at 37 °C. The absorbance at 490 nm was measured with a Bio-Rad 680 Microplate Reader (Bio-Rad, Hercules, CA, USA). All measurements were performed in triplicate. The experiments were performed independently three times. The IC_50_ was calculated using GraphPad PRISM (GraphPad PRISM Software 6.0, San Diego, CA, USA).

## 4. Conclusions

In this study, we designed and synthesized a series of mono-aliphatic chain-modified scutellarein derivatives, and evaluated their biological activity in inhibiting cancer cell proliferation. We identified scutellarein derivatives **5a**, **5b**, **6a** and **6b** as new anti-proliferation agents for Jurkat, HCT-116 and MDA-MB-231 cells. The results showed that a proper long aliphatic chain enhanced the anti-proliferative activity of scutellarein. For our further studies, compound **6a** was selected as a promising lead in order to generate more effective anticancer agents. 

## Figures and Tables

**Table 1:** In vitro antiproliferative activities of compounds.

Compound	IC_50_ Values (μM)		
	Jurkat	HCT-116	MDA-MB-231
**NaAsO_2_**	4.70 ± 0.44	31.20 ± 3.35	8.57 ± 1.13
**1**	156.02 ± 7.21	>200	>200
**2**	95.77 ± 2.56	>200	>200
**3**	181.06 ± 6.21	>200	>200
**4**	>200	>200	>200
**5a**	14.79 ± 2.69	22.02 ± 2.27	59.93 ± 4.46
**5b**	22.47 ± 1.70	55.56 ± 7.21	68.54 ± 9.18
**5c**	>200	>200	>200
**5d**	76.23 ± 9.85	184.06 ± 7.31	>200
**6a**	1.80 ± 0.49	11.50 ± 2.00	53.91 ± 4.04
**6b**	19.12 ± 0.70	53.53 ± 8.25	145.01 ± 10.03
**6c**	>200	>200	>200
**6d**	>200	>200	>200

## References

[1] Hsiao W., Liu L. (2010). The role of traditional Chinese herbal medicines in cancer therapy–from TCM theory to mechanistic insights. Planta Med..

[2] Sucher N.J. (2013). The application of Chinese medicine to novel drug discovery. Expert Opin. Drug Discov..

[3] Efferth T., Li P.C., Konkimalla V.S., Kaina B. (2007). From traditional Chinese medicine to rational cancer therapy. Trends Mol. Med..

[4] Gao J., Chen G., He H., Liu C., Xiong X., Li J., Wang J. (2017). Therapeutic Effects of Breviscapine in Cardiovascular Diseases: A Review. Front. Pharmacol..

[5] Qiu F., Xia H., Zhang T., Di X., Qu G., Yao X. (2007). Two major urinary metabolites of scutellarin in rats. Planta Med..

[6] Chan J.Y., Tan B.K.H., Lee S.C. (2009). Scutellarin Sensitizes Drug-evoked Colon Cancer Cell Apoptosis through Enhanced Caspase-6 Activation. Anticancer Res..

[7] Cheng C.-Y., Hu C.C., Yang H.J., Lee M.C., Kao E.S. (2014). Inhibitory Effects of Scutellarein on Proliferation of Human Lung Cancer A549 Cells through ERK and NFκB Mediated by the EGFR Pathway. Chin. J. Physiol..

[8] Li H.X., Huang D.Y., Gao Z.X.Z., Chen Y., Zhang L.N., Zheng J.H. (2013). Scutellarin inhibits the growth and invasion of human tongue squamous carcinoma through the inhibition of matrix metalloproteinase-2 and -9 and αvβ6 integrin. Int. J. Oncol..

[9] Han T., Li J., Xue J., Li H., Xu F., Cheng K., Li D., Li Z., Gao M., Hua H. (2017). Scutellarin derivatives as apoptosis inducers: Design, synthesis and biological evaluation. Eur. J. Med. Chem..

[10] Shi Z.H., Li N.G., Shi Q.P., Zhang W., Dong Z.X., Tang Y.P., Zhang P.X., Gu T., Wu W.Y., Fang F. (2015). Synthesis of scutellarein derivatives to increase biological activity and water solubility. Bioorg. Med. Chem..

[11] Dong Z.X., Shi Z.H., Li N.G., Zhang W., Gu T., Zhang P.X., Wu W.Y., Tang Y.P., Fang F., Xue X. (2016). Design, Synthesis, and Biological Evaluation of Scutellarein Derivatives Based on Scutellarin Metabolic MechanismIn Vivo. Chem. Biol. Drug Des..

[12] Song S., Li N., Tang Y., Wang Z., Qian L., Tang H., Duan J. (2012). Design, Synthesis and Biological Evaluation of Scutellarein Derivatives as Potential Anti-Alzheimer’s Disease Candidates Based on Metabolic Mechanism. Lett. Drug Des. Discov..

[13] Zhanga W., Dong Z.X., Gua T., Li N.G., Wu W.Y., Zhang P.X., Tang Y.P., Yang J.P., Fanga F., Li H.M. (2015). An improved synthesis of 6-*O*-methyl-scutellarein through selective benzylation. J. Chem. Res..

[14] Lin H., Zhang W., Dong Z.X., Gu T., Li N.G., Shi Z.H., Kai J., Qu C., Shang G.X., Tang Y.P. (2015). A New and Practical Synthetic Method for the Synthesis of 6-*O*-Methyl-scutellarein: One Metabolite of Scutellarin In Vivo. Int. J. Mol. Sci..

[15] Shi Z.H., Li N.G., Wang Z.J., Tang Y.P., Dong Z.X., Zhang W., Zhang P.X., Gu T., Wu W.Y., Yang J.P. (2015). Synthesis and biological evaluation of methylated scutellarein analogs based on metabolic mechanism of scutellarin in vivo. Eur. J. Med. Chem..

[16] Mo T., Liu X., Liu Y., Wang X., Zhang L., Wang J., Zhang Z., Shi S., Tu P. (2016). Expanded investigations of the aglycon promiscuity and catalysis characteristic of flavonol 3-*O*-rhamnosyltransferase from Arabidopsis thaliana. RSC Adv..

[17] Kong L.R., Chua K.N., Sim W.J., Ng H.C., Bi C., Ho J., Nga M.E., Pang Y.H., Ong W.R., Soo R.A. (2015). MEK Inhibition Overcomes Cisplatin Resistance Conferred by SOS/MAPK Pathway Activation in Squamous Cell Carcinoma. Mol. Cancer Ther..

[18] Li X., Li L., Huang Y., Liu B., Chi H., Shi L., Zhang W., Li G., Niu Y., Zhu X. (2017). Synergistic therapy of chemotherapeutic drugs and MTH1 inhibitors using a pH-sensitive polymeric delivery system for oral squamous cell carcinoma. Biomater. Sci..

[19] Dilda P.J., Hogg P.J. (2007). Arsenical-based cancer drugs. Cancer Treat. Rev..

